# Immunity to Ocular and Genital Herpes Simplex Viruses Infections

**DOI:** 10.1155/2012/732546

**Published:** 2012-12-26

**Authors:** Aziz Alami Chentoufi, Lbachir BenMohamed, Philippe Van De Perre, Ali A. Ashkar

**Affiliations:** ^1^Pathology and Clinical Laboratory Medicine, Department of Immunology, King Fahad Medical City, Riyadh 11525, Saudi Arabia; ^2^Laboratory of Cellular and Molecular Immunology, Gavin Herbert Eye Institute, College of Medicine, University of California, Irvine, CA 92697-4375, USA; ^3^INSERM U 1058, Université Montpellier 1, 34090 Montpellier, France; ^4^Department of Pathology and Molecular Medicine, McMaster Immunology Research Centre, McMaster University, Room MDCL4015, 1280 Main Street West, Hamilton, ON, Canada L8S 4K1

Herpes simplex virus type 1 and type 2 are amongst the most common human viral infection. Despite decades of research, currently, there is no vaccine or sterilizing therapy against human herpes. Both innate and adaptive immunities are required for protection against herpes infections. However, the immunity and immunopathology of herpes infections are not yet fully characterized. To better understand the immunopathology of herpes infections and ultimately design an efficient prophylactic or therapeutic herpes vaccine or treatment, it is fundamental to define the cellular and molecular immune mechanisms and the immune correlates required for an efficient control of these ubiquitous pathogens. Over 400,000 Americans suffer from ocular herpes caused by HSV-1. Each year, nearly 50,000 new and recurring ocular herpes cases are diagnosed in the United States, with the more serious stromal keratitis accounting for about 25 percent. About 1 billion people—one-sixth of the world's population—are infected with HSV-2, the most common cause of genital herpes. In the United States, an estimated 50 million people carry the virus, and up to 3 million of those people suffer recurrent outbreaks of painful genital herpes. While the majority of HSV-1 and HSV-2 seropositive individuals is asymptomatic, a nonnegligible number of symptomatic individuals have as often as four recurrent herpes per year and required treatments. In addition, genital herpes has played an important role in driving the prevalence of other sexually transmitted infection such as HIV. Current drug therapies, often used to suppress genital herpes, can also treat ocular herpes but do not prevent future outbreaks. There is currently no prophylactic or therapeutic vaccine against herpes in the market. The impairment of T-cell function in acute and latent infection has been reported at many levels including abnormal antigen presentation in the periphery and in the sensory ganglia (trigeminal and lumbosacral ganglia), latently infected neuronal cells resistance to apoptosis by CD8+ T-cells, unbalanced immunoregulatory T-cell function, and deregulation of Th1/Th2/Th17 axes. Despite the identification of many HSV-1 and HSV-2-antigens and their derived CD4+ and CD8+ T-cell epitopes, numerous antigen-specific therapies have failed when evaluated for their utility in the prevention and treatment of HSV infection. In this special issue, we invited authors to submit original research and review articles highlighting the recent advances that have broadened our understanding of immunity and immunopathology of herpes infection and HSV vaccine strategies. Also, we welcomed papers that seek to define immunoregulatory and effector properties of T-cells and dendritic cells to provide new insights as to their potential for clinical use. We were interested in articles that explore salient aspects of protective immunity throughout HSV latency, reactivation, ocular and genital herpetic disease, negative immunosynergy between HSV and HIV or other sexually transmitted diseases, and immunotherapeutical approaches.

 In a paper of this special issue, P. Stuart and T. Keadle documented the numerous recurrent models of herpetic stromal keratitis (HSK) that have been developed and how data generated from these models differs in some important aspects from data generated following primary infection of the cornea. Both differences and similarities have been described in these models with an eye towards possible vaccines and therapies that demonstrate promise in treating HSK.

 In another paper, the group of Y. Li identified the immunodominant epitopes (B-cell epitopes) in glycoproteins B (gB2), C (gC2), E (gE2), G (gG2), and I (gI2) of HSV-2 through software algorithms and experiments. The results suggested that the immunodominant epitopes screened using software algorithms may be used for virus diagnosis and vaccine design against HSV-2.

 A paper by K. Wu and coworkers highlighted the recent findings and controversies regarding the miRNA in herpes infection and immunity and demonstrated that HSV-1 miR-H6 inhibits HSV-1 replication and IL-6 expression in human corneal epithelial cells in vitro. It has been previously demonstrated that miR-H6 encoded from HSV-1 genome targets ICP4 to help maintain latency. In that study, synthesized miR-H6 mimics were transfected into HSV-1 infected human cornea epithelial cells (HCE). The inhibition of HSV-1 replication and viral ICP4 expression in miR-H6-transfected HCE was demonstrated. They also showed that miR-H6 decreased the interleukin 6 expression. The mechanism may provide an approach to prevent HSV-1 lytic infection and inhibit corneal inflammation.

 In another paper, S. Kopp et al. investigated how HSV-2 glycoprotein interacts with HVEM and influences virus-specific recall cellular responses at the mucosa. Infection of susceptible cells by HSV requires the interaction of the HSV gD glycoprotein with one of two principal entry receptors, herpes virus entry mediator (HVEM), or nectins. HVEM naturally functions in immune signaling, and the gD-HVEM interaction alters innate signaling early after mucosal infection. The study investigated whether the gD HVEM interaction during priming changes lymphocyte recall responses in the murine intravaginal model and conclude that engagement of HVEM during the acute phase of HSV infection influences the antiviral CD8+ recall response by a yet to be determined mechanism.

 In another paper, V. Tiwari and D. Shukla demonstrated how nonprofessional phagocytosis facilitated herpesvirus entry into ocular cells. Phagocytosis is a major mechanism by which the mediators of innate immunity thwart microbial infections. They demonstrate that human herpesviruses may have evolved a common mechanism to exploit a phagocytosis-like entrapment to gain entry into ocular cells. Using laser scanning confocal microscopy they show that successful infection of ocular cell types by HSV-1, CMV, or HHV-8 viruses, belonging to three divergent subfamilies of herpesviruses, is facilitated by induction of F-actin rich membrane protrusions. Inhibitors of F-actin polymerization and membrane protrusion formation, cytochalasin D and latrunculin B, were able to block infection by all three viruses. Similar inhibition was seen by blocking phosphoinositide 3 kinase signaling, which is required for microbial phagocytosis. Transmission electron microscopy data using human corneal fibroblasts for HSV-1, human retinal pigment epithelial cells for CMV, and human conjunctival epithelial cells for HHV-8 are consistent with the possibility that pseudopod-like membrane protrusions facilitate virus uptake by the ocular cells. The study suggests a novel mechanism by which the innate mediators of phagocytosis can be infected by human herpesviruses.

 In another paper, K. Bryant-Hudson and D. J. J. Carr demonstrated how Programmed Death 1 (PD-1) and its ligands (PD-L1/PDL2) expressed on dendritic cells contributed to viral resistance during acute HSV-1 infection. The contribution of the receptor/ligand pair during an acute infection is less understood. HSV-1 infected mice administered neutralizing antibody to PD-L1 or PD-L2 were assessed for viral burden and host cellular immune responses. Virus titers were elevated in cornea and trigeminal ganglia (TG) of anti-PD-L1 treated mice, which corresponded with a reduced number of CD80-expressing dendritic cells, PD-L1+ dendritic cells, and HSV-1- specific CD8+ T cells within the draining (mandibular) lymph node (MLN). In contrast, anti-PD-L2 treatment had no effect on viral replication or changes in the MLN population. Notably, analysis of CD11c-enriched MLN cells from anti-PDL1 treated mice revealed impaired functional capabilities. The study suggested PD-L1-expressing dendritic cells are important for antiviral defense during acute HSV-1 infection.

 In another paper by A. A. Chentoufi et al., the authors specifically went over the old, the new, and the unknown issues related to herpes vaccine development, with specific emphasis on past, present, and future clinical trials. In spite of several clinical trials, starting as early as 1920s, no vaccine has been proven sufficiently safe and efficient to warrant commercial development. In recent years, great strides in cellular and molecular immunology have stimulated creative efforts in controlling herpes infection and disease. They describe how it is necessary to answer two fundamental questions: (i) Why past herpes vaccines have failed? (ii) Why the majority of HSV seropositive individuals (i.e., asymptomatic individuals) are naturally “protected” exhibiting few or no recurrent clinical disease, while other HSV seropositive individuals (i.e., symptomatic individuals) have frequent ocular, orofacial, and/or genital herpes clinical episodes? The group recently discovered several discrete sets of HSV-1 and HSV-2 symptomatic and asymptomatic epitopes identified by CD4+ and CD8+ T cells from seropositive symptomatic versus asymptomatic individuals. These symptomatic and asymptomatic epitopes will provide a solid foundation for the development of a future mucosal “asymptomatic” prime-boost vaccine that could optimize local protective immunity.

Finally, A. A. Chentoufi and L. BenMohamed have reviewed the phenotypic and functional properties of innate and adaptive mucosal immunity; their role in antiherpes immunity and immunopathology is reviewed. The progress and limitations in developing a safe and efficient mucosal herpes vaccine are discussed.

*Aziz Alami Chentoufi* *Lbachir BenMohamed* *Philippe Van De Perre* *Ali A. Ashkar*

